# Structure-Based Design and Synthesis of a New Phenylboronic-Modified Affinity Medium for Metalloprotease Purification

**DOI:** 10.3390/md15010005

**Published:** 2016-12-27

**Authors:** Shangyong Li, Linna Wang, Ximing Xu, Shengxiang Lin, Yuejun Wang, Jianhua Hao, Mi Sun

**Affiliations:** 1 Key Laboratory of Sustainable Development of Marine Fisheries, Ministry of Agriculture, Yellow Sea Fisheries Research Institute, Chinese Academy of Fishery Sciences, 106 Nanjing Road, Qingdao 266071, China; lshywln@163.com (S.L.); wlnwfllsy@163.com (L.W.); wangyj@ysfri.ac.cn (Y.W.); haojh@ysfri.ac.cn (J.H.); 2 Institute of Bioinformatics and Medical Engineering, School of Electrical and Information Engineering, Jiangsu University of Technology, Changzhou 213000, China; ximing.xu@jsut.edu.cn; 3 Laboratory of Oncology and Molecular Endocrinology, CHUL Research Center (CHUQ) and Laval University, 2705 Boulevard Laurier, Ste-Foy, Ville de Québec, QC G1V 4G2, Canada; 4 Laboratory for Marine Drugs and Bioproducts, Qingdao National Laboratory for Marine Science and Technology, Qingdao 266237, China

**Keywords:** metalloprotease, adsorption analysis, molecular docking, affinity purification, aminophenylboronic acid

## Abstract

Metalloproteases are emerging as useful agents in the treatment of many diseases including arthritis, cancer, cardiovascular diseases, and fibrosis. Studies that could shed light on the metalloprotease pharmaceutical applications require the pure enzyme. Here, we reported the structure-based design and synthesis of the affinity medium for the efficient purification of metalloprotease using the 4-aminophenylboronic acid (4-APBA) as affinity ligand, which was coupled with Sepharose 6B via cyanuric chloride as spacer. The molecular docking analysis showed that the boron atom was interacting with the hydroxyl group of Ser176 residue, whereas the hydroxyl group of the boronic moiety is oriented toward Leu175 and His177 residues. In addition to the covalent bond between the boron atom and hydroxyl group of Ser176, the spacer between boronic acid derivatives and medium beads contributes to the formation of an enzyme-medium complex. With this synthesized medium, we developed and optimized a one-step purification procedure and applied it for the affinity purification of metalloproteases from three commercial enzyme products. The native metalloproteases were purified to high homogeneity with more than 95% purity. The novel purification method developed in this work provides new opportunities for scientific, industrial and pharmaceutical projects.

## 1. Introduction

Proteases are enzymes that catalyze the hydrolysis of peptide bonds. Based on the mechanism of catalysis, proteases can be classified into six classes, including metallo, serine, aspartic, cysteine, glutamic, and threonine proteases [1]. Proteases are the most important industrial enzymes, accounting for more than 60% of the total enzyme market [2,3]. They have broad applications in the pharmaceutical, leather, food, and detergent industries [1,2,3,4]. Proteases play critical roles in normal biological processes; their unusual activities have been implicated in the development and progression of many diseases, e.g., fibrosis, arthritis, cancer, cardiovascular diseases, nephritis, and central nervous system disorders [5,6,7]. Among all of the six classes of proteases, only untagged serine proteases can be purified in one step using *p*-aminobenzamidine-modified affinity medium [8,9]. This simple procedure of affinity purification significantly accelerated the pharmaceutical application of many serine proteases [10,11,12,13,14,15].

Currently, there is no straightforward and efficient protocol for the purification of metalloproteases [14,15,16]. The traditional protocol that has multiple steps is expensive and results in low recovery [17,18,19,20]. Although some reports refer to the high-yield purification of metalloproteases (more than 90% purity) in one-step procedure, these protocols were based on immobilized metal affinity chromatography (IMAC) that has its disadvantages [21,22]. The first one is the use of high concentrations of imidazole and salt in the elution buffer of the IMAC procedure, which necessitates additional dialysis or a desalting step [23,24]. Also, it is well known that purification of a metalloprotein via a metal ion chelated by the resin in a similar manner results in the exchange of metal ion from resin with a metal ion from metalloprotein. This metal transfer causes a decrease in the stability of purified metalloprotein [21]. In addition, the use of chelating agents during purification has to be avoided as these compounds can remove the metal ion from the enzyme active site [23,24,25]. Therefore, design, synthesis and application of a new specific and efficient medium for the purification of metalloproteases are important tasks.

The structure-based design of the affinity ligand that serves as a specific inhibitor or substrate analogue is an efficient and commonly used approach in the affinity purification of enzymes [26,27,28]. An alkaline metalloprotease, MP (accession no. ACY25898) from marine bacterium *Flavobacterium* sp. YS-80-122, has been previously isolated in our laboratory [3]. This enzyme is a typical Zn-containing metalloprotease with antioxidant activity, and it has been commercially used as a detergent additive. The analysis of its crystal structure (PDB: 3U1R) [29] allowed suggestion of a novel affinity ligand that could reversibly bind to the active site and could be used for the affinity purification of the enzyme. Our preliminary virtual screening and experimental verification indicated that boronic acid derivatives (BADs) could reversibly inhibit the activity of MP [30]. Phenylboronate group, which can form a temporary covalent bond with any molecule that contains a 1,2-*cis*-diol group, is widely used in the affinity purification of 1,2-*cis*-diol-containing biomolecules such as glycoproteins, glycopeptides, nucleosides, and nucleic acids [31,32,33,34,35]. However, application of the resins modified by phenylboronate in the purification of metalloproteases has never been reported.

Here, the phenylboronate-modified resin was synthesized through the coupling of 4-aminophenylboronic acid (4-APBA) with epoxy-activated Sepharose 6B via cyanuric chloride spacer. The binding site and structure-activity relationship between 4-APBA-modified medium and MP were analyzed using molecular docking and adsorption determination, correspondingly. The synthesized medium was used for development of one-step affinity purification of metalloproteases. Three commercially available metalloproteases were efficiently purified with a high purity (more than 95%) using the protocol developed. Our research provides new opportunities for the development of industrial methods of metalloprotease purification.

## 2. Results and Discussion

### 2.1. Design and Synthesis of Affinity Medium for Metalloprotease Purification

Our initial virtual screening showed that some BADs could inhibit MP catalytic activity [30]. To confirm that BADs could inhibit MP, ten BADs were purchased or synthesized, and then their inhibitory effect was tested on MP. Surprisingly, three compounds were strong MP inhibitors with apparent *K*_i_ value of 0.8–1.2 μM. Thus, we focused our efforts on the design of BADs-based affinity medium for metalloprotease purification. Immobilisation of a ligand onto the epoxy-activated resin should be achieved via a nucleophilic group present in the ligand, often a primary amine [27]. Aminephenylboronic acid was chosen as an affinity ligand for our study because it was commercially available and had a favorable configuration for synthesis affinity medium.

**Figure 1:**
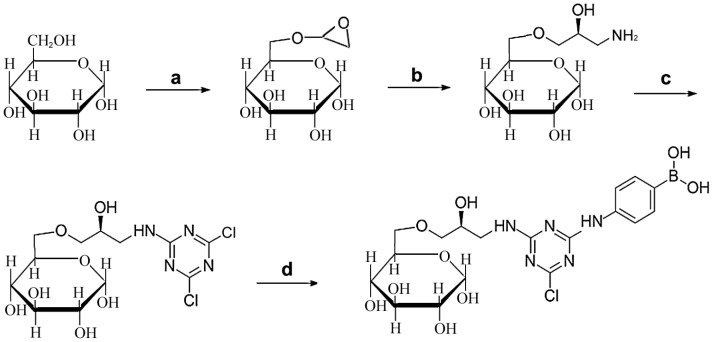
Synthesis protocol and scheme of the 4-APBA ligand coupled with actived Sepharose 6B via cyanuric chloride spacer. Reagents and conditions: (**a**) epichlorohydrin, DMSO, NaOH aqueous solution, 2.5 h; (**b**) 35% saturated ammonia, overnight; (**c**) cyanuric chloride, 50% acetone, pH 7–8; (**d**) 4-APBA, sodium carbonate, 24 h.

The nature of the immobilized complex or, in other words, the choice of affinity ligand and spacer arm, has a major influence on the outcome of a biomimetic affinity of purification procedure [27,28,29,36]. To obtain an optimal affinity medium, two types of APBA-based ligands, 4-APBA, and 3-APBA, were tested. The affinity ligands were coupled with activated Sepharose 6B via cyanuric chloride spacer. To estimate the effect of the presence of a boron atom in the affinity ligand, another type of the affinity ligand lacking of boron atom (aniline ligand) was synthesized. The scheme for the synthesis of 4-APBA-modified Sepharose 6B is shown in Figure 1. To confirm the ligand structure, the medium was hydrolyzed with 6 M HCl, and then the resultant with molecular formula of C12H15BClN5O4 and molecular mass of 339.5. Because the chlorine on the triazine ring was unstable in acidic condition, the hydrolysis with 6 M HCl would replace the chlorine on the ligand with a hydroxyl group [37], thus the theoretical structure of the purified ligand should be with a molecular formula of C12H16BN5O5 and molecular mass of 321.1. The ligand may be broken into fragments as C9H10BN5O3 at cone voltage of 170 V and molecular mass of 247.01. As shown in Figure S1, the main peak, 247.02, showed good agreement with [M-C3H6O2-H]^+^. The possible structures of chemicals in principal peaks are also shown in Figure S1. These results showed that the synthesized ligands had a good reliability.

**Figure 2:**
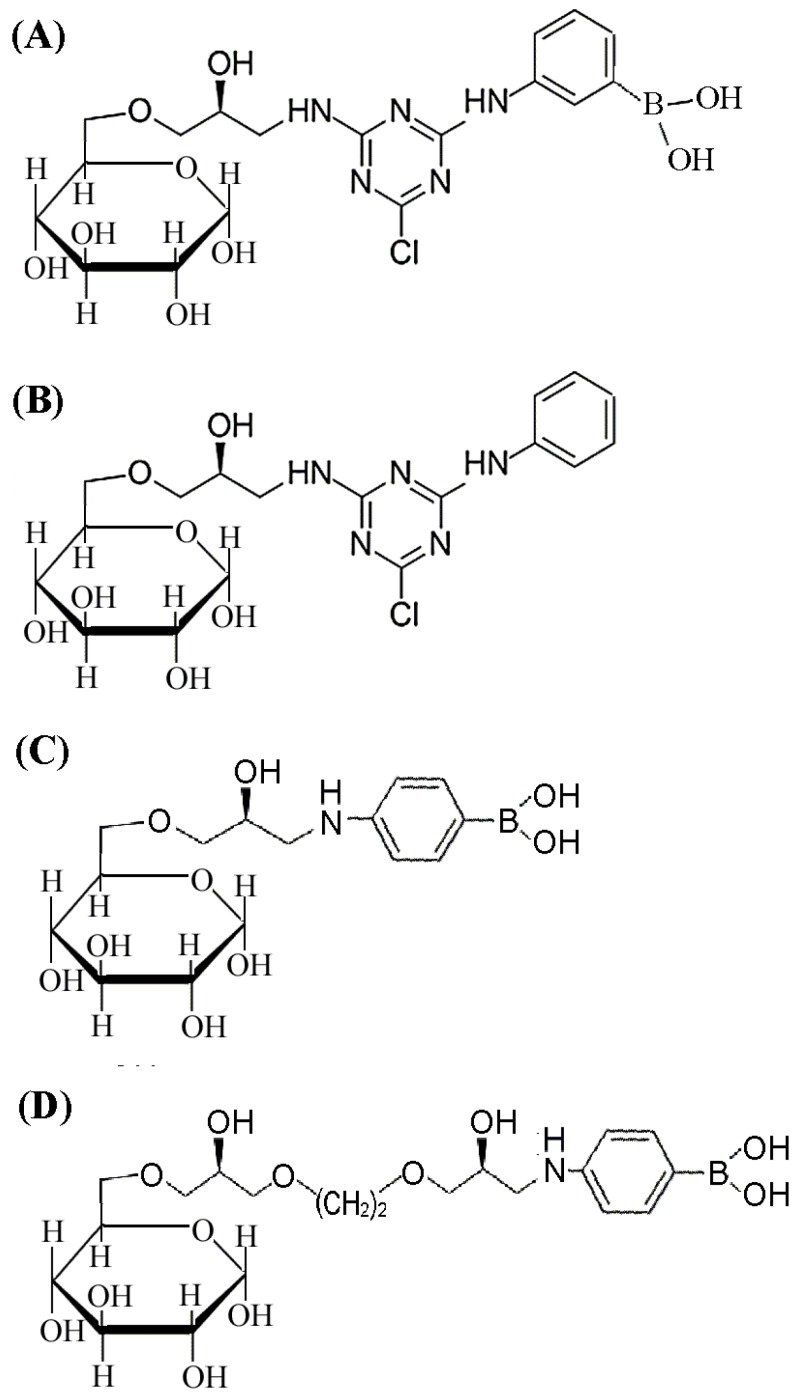
The scheme of four different affinity media. (**A**) 3-APBA ligand coupled with activated Sepharose 6B via cyanuric chloride spacer; (**B**) Aniline ligand coupled with activated Sepharose 6B via cyanuric chloride spacer; (**C**) 4-APBA ligand coupled with activated Sepharose 6B via 5-atom spacer arm; (**D**) 4-APBA ligand coupled with activated Sepharose 6B via 10-atom spacer arm.

**Figure 3:**
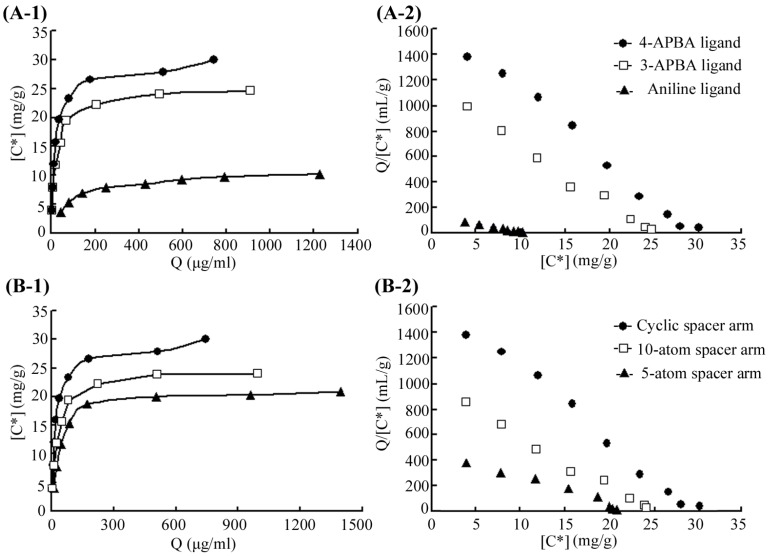
Adsorption analyses of different affinity media. (**A**) Adsorption analysis of affinity media with three different ligands via the same spacer arm (cyanuric chloride); (**B**) Adsorption analysis of affinity media with the same ligand (4-APBA) via three different spacer arms. (**1**) Equilibrium adsorption of metalloprotease (MP) on the affinity medium in a batch system (50 mM Gly-NaOH buffer, pH 8.6, 25 °C), (**2**) Plot describing the equilibrium of the absorption on the medium and the enzyme concentration in the liquid phase.

The 3-APBA-modified medium and aniline-modified medium were synthesized using the same concentration of 3-APBA or aniline as for 4-APBA (Figure 2A,B). The density of the free amino groups was determined by the ninhydrin test before the adding of the APBA ligands, giving equal ligand densities (Table 1). Equilibrium adsorption studies were performed to characterize the affinity value of MP and these three affinity media (Figure 3A). Desorption constant for the 4-APBA-modified medium was 14.9 μg/mL which was significantly lower than that for the 3-APBA medium (21.5 μg/mL) and aniline medium (67.2 μg/mL). Meanwhile, the theoretical maximum absorption (*Q*_max_) for the 4-APBA medium (29.6 mg/g) was significantly higher than it was for the other two media (24.9 mg/g and 10.6 mg/g, respectively) (Table 1), indicating the high affinity of 4-APBA-modified Sepharose 6B towards MP. Therefore, 4-APBA was chosen as the affinity ligand for the further design and synthesis of affinity medium.

To find the optimal spacer arm, two different lengths of linear arms (5-atom spacer and 10-atom spacer) and a cyclic arm (cyanuric chloride) were tested. Cyanuric chloride is a typical cyclic compound containing the *s*-triazine (C_3_N_3_) ring that could supply a higher mechanical strength for the ligand stabilization and was widely used in the affinity medium synthesis [38,39,40,41]. The scheme for the synthesis of media with the 5-atom spacer and the 10-atom spacer are shown in Figure 2C,D, correspondingly. In the adsorption analysis (Figure 3B), 4-APBA ligand with cyclic spacer arm exhibited the highest adsorption value, even though its epoxy content (20.9 μmol/mL) was lower than the content of 5-atom linear spacer (41.8 μmol/mL) and the 10-atom linear spacer (27.8 μmol/mL) (Table 1). Thus, cyanuric chloride was chosen as the compound for generation of optimal spacer arm.

### 2.2. Binding Analysis for 4-APBA-Modified Medium and MP

**Figure 4:**
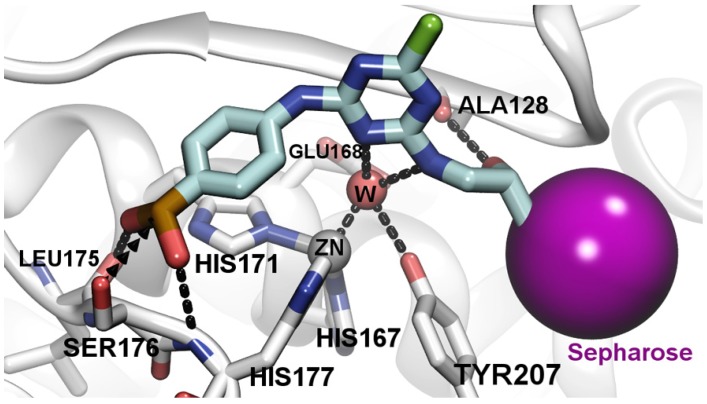
The binding mode of MP and the 4-APBA-modified medium. The atom force field maps were generated using Autogrid4 software for AutoDock4 (Zn); binding conformation was analyzed by Lamarckian Genetic Algorithm-Local Search combined algorithm with default searching parameter.

Quite a few of studies show that boron-containing small molecules interacted with proteins through a covalent bond between the boron atom and the oxygen atom in the hydroxyl group of a serine [42]. In this study, the molecular docking analysis also indicated that the boron atom interacted with the hydroxyl group of Ser176 residue through covalent bonding, whereas the hydroxyl group of the boronic moiety is oriented toward Leu175 and His177 residues (Figure 4). We found that several secondary interactions could contribute to the stabilization of MP interaction with 4-APBA-modified medium. For example, the benzene ring of the 4-APBA ligand formed a π-π interaction with His171 residue of MP. In addition, the hydrogen bond between the *s*-triazine ring of the spacer and the molecule of water was observed, as well as the hydrogen bond between the hydroxyl group of an atom of the Ala128 residue. The aniline ligand bound with Sepharose 6B via cyanuric chloride also exhibited a low affinity (*K*_d_, 67.2 μg/mL; *Q*_max_, 10.6 mg/g) toward MP, implying that several secondary interactions can occur in addition to the interaction with the boronate ion.

Boronate affinity materials have gained increasing attention in recent years [31,32,33]. The mechanism involved is similar to other conventional boronate affinity chromatography. Moreover, other possible binding mechanisms were also exhibited in the molecular docking performance. One performance showed that it could be possible for Ser176 and His177 to interact with the hydroxyl groups of the boronic acid (not the boron atom) through hydrogen binding [31]. This binding mechanism relied on the hydrogen binding, which exhibited much lower affinity than the conventional binding. In the adsorption analysis, the aniline ligand exhibited a much lower affinity than APBA ligand with boronic acid, implying that the boronic acid was very important in the binding mechanism. The other possible performance is for the boron atom to coordinate with the water molecule through intermolecular B-N coordination [34]. The Ser176 residue was located in the bottom of the active-site pocket that had enough space for binding with a molecule larger than the molecule of water. Also, the 4-APBA ligand with 10-atom linear spacer showed a similar adsorption value with that for the 5-atom spacer, even though its epoxy content (27.8 μmol/mL) was smaller than the 5-atom spacer (41.8 μmol/mL) (Table 1). This probably occurred because the longer spacer arm provided the larger spatial distance and thus provided a better accessibility of the Ser176 residue in the cavity of active site. Summarizing, the boron atom bound to MP by trapping the Ser176 hydroxyl group in the active site pocket.

### 2.3. One-Step Affinity Purification of Commercial Metalloprotease Products

Three commercially available products (MP, DENIE-B LPS-P and ViscozymeL) containing metalloproteases were dissolved in the loading buffer to a final concentration of 10 mg/mL each. Then, the enzymes were purified by a one-step purification protocol using the 4-APBA-modified Sepharose 6B medium. We tested different loading and elution conditions to optimize the yield of metalloproteases. Almost all of the metalloproteases contained seven or eight calcium ions stabilizing their three-dimensional structure [29]. Thus, to obtain properly folded enzymes, both of the loading and elution buffers contained 1 mM CaCl_2_. The 0.1 M Gly-NaOH buffer, pH 8.6, was chosen as the loading buffer because of the highest affinity of MP to the beads and stability of all three enzymes at this pH. Different acetic acid buffers (pH ranging from 4.0 to 6.0) were tested to select an optimal pH for MP elution, as low acidity favored the disruption of the H-bond interactions between MP and the medium. The highest protein yield was obtained at pH 5.4. Thus, 0.1 M acetic acid (pH 5.4) was chosen as the elution buffer. The SDS-PAGE analysis of the crude and purified metalloproteases is shown in Figure S2. The activity and purity of purified enzymes are shown in Table 2.

In our previous work, the five-step purification protocol for the purification of MP was developed. It included ammonium sulfate precipitation, desalting, anion-exchange and gel-filtration chromatography and took more than 48 h of work [3,21]. Here, we report a simple and efficient one-step MP purification procedure that takes less than one hour. Our protocol is based on the 4-APBA-modified Sepharose 6B medium that efficiently bound native MP from natural sources. Here we compared this one-step protocol with the previous reported methods, along with all the different purification steps, activity yields, specific activities and time requirement. According to the measurements of MP activity in the initial sample and purified protein, almost 64.1% of initial MP was purified, whereas only 8.9% of initial MP was recovered using the traditional protocol. The specific activity of MP purified by the APBA-modified protocol (95.6 U/mg) is similar with the value obtained through the traditional purification protocol (96.2 U/mg) and the IMAC protocol (94.8 U/mg). However, the purity of MP is different, being higher with the APBA-modified affinity purification (98.8%) with respect to the traditional and IMAC methods (92.5% and 94.7%, respectively). Even if the IMAC protocol results an activity recovery higher than the 4-APBA protocol, it is longer. Moreover, the APBA-modified affinity protocol avoids the use of toxic imidazole and the loss of metallic ions in the MP active pocket that reduce the enzyme stability. Based on all the positive features of this affinity protocol, such as one-step of chromatography, shorter times, and higher purity, it is clear there is potential in this approach for the industrial production of high-purity MP.

**Figure 5:**
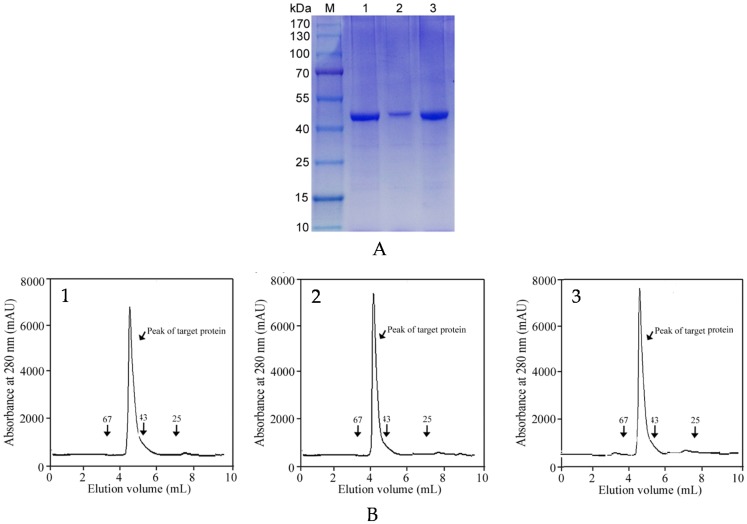
Purity analysis of three purified enzyme products. (**A**) SDS-PAGE (10.0%) analysis showed that the enzymes were purified to an apparent homogeneous population with a molecular mass of 48 kDa and the purity was more than 95%. *Lane M*, molecular mass standard protein marker; *Lane 1*, the purified MP; *Lane 2*, the purified DENIE-B LPS-P; *Lane 3*, the purified ViscozymeL; (**B**) HPLC analysis using the size exclusion by gel filtration of the purified MP (**1**), DENIE-B LPS-P (**2**) and ViscozymeL (**3**) on a TSK 3000SW column.

To determine whether our medium has an affinity value to metalloproteases from other sources, two other commercial metalloprotease products, DENIE-B LPS-P and ViscozymeL, were used for enzyme purification. DENIE-B LPS-P was an enzyme concentrate produced from *Bacillus subtilis* that was widely used in leather softening [43]. Based on the activity measurement in our study, only 10.7% of metalloprotease was purified from this commercial product using the traditional three-step purification protocol, including ammonium sulfate precipitation, desalting and anion-exchange chromatography on a Q Sepharose column. ViscozymeL was a cell wall degrading enzyme complex from *Aspergillus* sp., containing a wide range of carbohydrases and metalloprotease [44]. Traditional purification of the metalloprotease from ViscozymeL resulted in only less than 60% pure enzyme, which required six steps, including ammonium sulfate precipitation, hydrophobic chromatography, desalting, anion-exchange chromatography, and two steps of gel-filtration chromatography. Meanwhile, IMAC (Cu-IDA ligand) purification of these two protein products resulted in less than 60% purity of metalloproteases (data not shown). However, the 4-APBA-modified medium could efficiently purify metalloproteases from those two products (Table 2). The activity recoveries of DENIE-B LPS-P and ViscozymeL were 45.2% and 37.8%, respectively. Meanwhile, when the purified enzymes were analyzed by HPLC with a TSK3000SW gel filtration column, both of them were more than 95% pure (Figure 5). To sum, our novel methodology had multiple advantages in comparison with all known techniques of metalloprotease purification.

## 3. Materials and Methods

### 3.1. Materials

The dried powders of crude metalloprotease, MP, were yielded from marine bacterium *Flavobacterium* sp. YS-80-122. A commercial metalloprotease concentrate produced from *Bacillus subtilis*, DENIE-B LPS-P, was purchased from Denykem Ltd. (Shanghai, China). Cell wall degrading enzyme complex from *Aspergillus* sp., ViscozymeL, containing a wide range of carbohydrases and metalloprotease was obtained from Novozymes, Denmark. The 4-aminophenylboronic acid, 3-aminophenylboronic acid (3-APBA), aniline and cyanuric chloride (2,4,6-trichloro-1,3,5-triazine) were purchased from Sigma-Aldrich, St. Louis, MO, USA. Activated Sepharose 6B with two different spacer arm lengths (5-atoms, 10-atoms) were from Beijing Weishibohui Chromatography Technology Co., Beijing, China. All remaining reagents were of analytical grade (Sinopharm Chemical Reagent, Shanghai, China).

### 3.2. Synthesis of Affinity Medium

The affinity media were prepared according to the methods developed previously [45,46]. The scheme of the synthesis procedure is shown in Figure 1. Initially, Sepharose 6B was modified by epichlorohydrin to form activated amino-sepharose. Briefly, Sepharose 6B (100 g) was thoroughly washed with deionized water at a 1:10 ratio until the pH value of the eluate reached 7.0 and the beads were dried. To activate Sepharose 6B, the beads were resuspended in 50 mL of activating solution (1 M NaOH, 2.5 g DMSO, and 10 mL epichlorohydrin) followed by incubation at 40 °C for 2.5 h with shaking (Figure 1a). Then, 35% saturated ammonia (150 mL) was added to the activated Sepharose 6B resuspended in 350 mL distilled water. The beads were incubated overnight at 30 °C on a rotary 39 shaker to form aminated Sepharose 6B (Figure 1b). To attach cyanuric chloride to the amino groups of aminated Sepharose 6B, the beads were resuspended in 350 mL 50% (*v*/*v*) acetone in an ice-salt bath, and then 8 g of cyanuric chloride dissolved in 70 mL acetone was added with a flow rate of 0.5 mL/min in the shaking station. The neutral pH was maintained by simultaneous addition of 1 M NaOH. The beads were washed with 50% (*v*/*v*) acetone to remove the free cyanuric chloride (Figure 1c). The density of the free amino group was determined by the ninhydrin test in the following procedure: a small aliquot of beads was smeared on filter paper, sprayed with ninhydrin solution (0.2% (*w*/*v*) in acetone), and heated briefly with a hair dryer. The appearance of purple color indicated the presence of free amino groups, whereas the color disappearance indicated that cyanuric chloride had been linked to the amino groups [27]. Then, a twofold excess of 4-APBA dissolved in 2 M sodium carbonate was added to the dichlorotriazinylated Sepharose 6B beads. After 24 h of stirring at room temperature, the beads were filtered, washed well with water and stored in 0.02% (*w*/*v*) sodium azide (Figure 1d). To confirm the conformation of the 4-APBA ligand on the medium, 100 mg dried medium was incubated with 6 M HCl at boiling condition for 24 h, and HCl was removed by vacuum evaporation. The hydrolyzed chemical was purified and analyzed with ESI-MS (HP1100LC MSD, Agilent, San Francisco, CA, USA) according to the methods reported [37].

To generate control beads with 3-APBA and/or aniline, the affinity medium with 3-APBA or aniline instead of 4-APBA was synthesized according to the described method above (Figure 2A,B). To generate control beads with two different spacer arms, the 4-APBA-modified Sepharose 6B beads with 5-atom or 10-atom spacer arms were synthesized according to the published method [21,28,36]. The schemes for the generation of these beads are shown in Figure 2C,D. Briefly, 5 g of 4-APBA dissolved in 80 mL of 2 M sodium carbonate was added to the previously activated Sepharose 6B. After 24 h of incubation at room temperature with stirring, the beads were filtered, washed well with water and stored in 0.02% (*w*/*v*) sodium azide [13,14].

### 3.3. Adsorption Value Analysis

To characterize the interaction of MP with five different types of affinity media, an equilibrium adsorption study was performed. The constant of desorption (*K*_d_) and the theoretical maximum adsorption capacity (*Q*_max_) of these affinity media were analyzed according to the Scatchard analysis model [24,47]. Briefly, one milliliter of increasing concentrations of purified metalloprotease (0.1–0.9 mg/mL in 20 mM Gly-NaOH buffer, pH 8.6) was mixed with 0.5 g of each affinity medium and shaken for 2 h at 4 °C until the solution reached adsorption equilibrium. Then, the mixtures were centrifuged at 1500 *g* for 5 min. The protease activity and protein concentration were measured in the supernatants.

The analysis of equilibrium adsorption provided a relationship between the concentration of metalloprotease in the solution and the amount of enzyme absorbed on the affinity medium. The data obtained were analyzed using the Scatchard plot according to the following equation:

$$Q=\frac{Q_{\max}\left[C^{*}\right]}{K_{d}+\left[C^{*}\right]}$$

Therein, *Q* is the adsorption amount of enzyme to the medium (mg/g), *Q*_max_ is the theoretical maximum of metalloprotease absorption to the affinity medium (mg/g), [C*] is the concentration of metalloprotease in solution (mg/mL), and *K*_d_ is the desorption constant.

### 3.4. Molecular Docking Analysis

The MP protein structure (PDB ID 3U1R) [26] was prepared by AutoDockTools (The Scripps Research Institute, San Diego, CA, USA). Briefly, hydrogens and gasteiger charge were added and waters were removed, except the water molecules bound to the zinc ion, which was treated as hydrogen acceptor. Ca^2+^ and Zn^2+^ were kept in the structure. The protein structure was then prepared according to the reference [48], using an improved zinc force field for AutoDock4 (Zn) (The Scripps Research Institute, San Diego, CA, USA). For the ligand, the sepharose part was not considered in molecular docking, as it was an inert polymeric support, and frequently used for coupling the “active” affinity ligands to the matrix. The ligand structure was built and minimized with Maestro (Schrödinger LLC., Cambridge, MA, USA). The type of boron atom was set to be *sp^3^* hybridization to mimic its binding with the hydroxyl group in Ser/Thr amino acids. Finally, the ligand was converted to the pdbqt format by AutoDockTools. The atom force field maps were generated using Autogrid4 software for AutoDock4 (Zn); binding conformation was searched by Lamarckian Genetic Algorithm-Local Search combined algorithm with default searching parameter. Fifty conformations were generated for further analysis. The representation was visualized with VMD 1.9.2 software (The Scripps Research Institute, San Diego, CA, USA) [49].

### 3.5. Traditional and Affinity Purification of Three Commercial Metalloproteases

Two grams of dried powder of three commercially available products containing three different metalloproteases, MP, DENIE-B LPS-P, and ViscozymeL, were dissolved in 50 mL sample loading buffer (0.1 M Gly-NaOH buffer, pH 8.6) each. The traditional purification protocol of MP was composed of five steps, including ultrafiltration, ammonium sulfate precipitation on 60% saturation, desalting, anion-exchange on a Q-sepharose column and gel-filtration chromatography on Sephacryl S-200 HR [3]. Meanwhile, the other two commercial metalloproteases were purified used traditional column purification protocol in this study. The traditional purification protocol of DENIE-B LPS-P was composed of three steps, including ammonium sulfate precipitation on 60% saturation, desalting and anion-exchange chromatography on a Q-sepharose column. The traditional purification protocol of ViscozymeL was composed of six steps, including ammonium sulfate precipitation on 40% saturation, hydrophobic chromatography on a phenyl column, desalting, anion-exchange chromatography on a diethylaminoethanol(DEAE)-sepharose column, and two step of gel-filtration chromatography on Sephacryl S-200 HR (GE Healthcare, Madison, WI, USA).

In the affinity purification protocol, the supernatant was loaded onto 10 mL pre-equilibrated column and washed with washing buffer (0.1 M Gly-NaOH buffer, pH 8.6) until the eluate exhibited no detectable absorbance at 280 nm. The target protein was eluted with elution buffer (0.1 M acetic acid buffer, pH 5.4). The flow rate of the mobile phase was 3.0 mL/min. The concentrations of each elution peak were measured by the Bradford method, using bovine serum albumin (BSA) as a standard. The purified enzyme was further characterized by 10% SDS-PAGE and high performance liquid chromatography (HPLC) analysis. The purification process was repeated more than five times.

### 3.6. Enzymatic Activity Assay

One hundred microliters of enzyme solution were mixed with 4.9 mL of casein solution (0.6% (*w*/*v*) in 25 mM borate buffer, pH 10.0) and incubated at 25 °C for 10 min. The relative enzyme activity was measured using Folin-Ciocalteu’s method [3,4]. One unit was defined as the amount of enzyme causing the release of 1 μg tyrosine per minute under the above conditions.

### 3.7. Protein Purity Analysis

SDS-PAGE analysis was carried out on a Mini-protean II system from Bio-Rad (Hercules, CA, USA). The purity of the purified proteases was calculated by a gel imaging analysis system (Gelpro Analyzer 3.2 (Thermo Fisher Scientific, Waltham, MA, USA) according to the integration of the lane darkness. HPLC (Agilent 1260, San Francisco, CA, USA) analysis was performed with a TSK3000SW gel filtration column (Tosoh Co., Tokyo, Japan) monitored at 280 nm [27]. The solvent phase was 0.1 M PBS, 0.1 M Na_2_SO_4_, 0.05% NaN_3_, pH 6.7. The flow rate was 0.6 mL/min.

## 4. Conclusions

In this study, an affinity medium more efficient for metalloprotease purification than other currently available techniques was designed, synthesized and experimentally characterized. Testing the adsorption properties of five designed resins, the Sepharose 6B media coupled with 4-APBA via cyanuric chloride spacer was selected for the purification of native metalloproteases from three commercial products. Metalloproteases from these sources were purified in one step with high efficiency and purity (more than 95%). Compared with the previously reported methods, this protocol resulted in several positive features, such as fewer steps, better activity recoveries, and higher purity. Coupled with efficacy, time-saving procedure and accessible reagents, this novel affinity purification protocol represents a potential important tool for industrial application.

## Figures and Tables

**Table 1:** Ligand densities, desorption constant (*K*_d_) and theoretical maximum absorption (*Q*_max_) analysis of the affinity media.

Ligands	Spacer Arms	Ligand Density (μmol/mL)	*K*_d_ (μg/mL)	*Q*_max_ (mg/g)
Aniline	Cyanuric chloride	20.9	67.2	10.6
3-APBA ^a^	Cyanuric chloride	20.9	21.5	24.9
4-APBA	Cyanuric chloride	20.9	14.9	29.6
4-APBA	10-atom spacer	41.8	24.4	24.6
4-APBA	5-atom spacer	27.8	46.3	22.3

^a^ APBA represents aminophenylboronic acid.

**Table 2:** Comparison of affinity and traditional purification methods for three available metalloprotease products.

Enzymes	Purification Method	Activity Recovery (%)	Protein Purity (%)	Specific Activity (U/mg)	Time Requirement
MP	Affinity protocol ^a^	64.1	98.8	95.6	~1 h
	Traditional protocol ^b^	8.9	97.6	96.2	>48 h
DENIE-B LPS-P	Affinity protocol ^a^	45.2	95.9	64.6	~1 h
	Traditional protocol ^c^	10.7	91.4	62.3	>48 h
ViscozymeL	Affinity protocol ^a^	37.8	97.1	51.4	~1 h
	Traditional protocol ^d^	3.4	58.3	27.8	>96 h

^a^ In the affinity protocol, enzymes were purified by 4-APBA-modified medium; ^b^ The traditional purification protocol of MP was composed of five steps, including ultrafiltration, ammonium sulfate precipitation, desalting, anion-exchange and gel-filtration chromatography; ^c^ The traditional purification protocol of DENIE-B LPS-P was composed of three steps, including ammonium sulfate precipitation, desalting and anion-exchange chromatography; ^d^ The traditional purification protocol of ViscozymeL was composed of six steps, including ammonium sulfate precipitation, hydrophobic chromatography, desalting, anion-exchange chromatography, and two steps of gel-filtration chromatography.

## Supplementary Materials

The following are available online at www.mdpi.com/1660-3397/15/1/5/s1. Figure S1: The ESI-MS analysis of the affinity ligand. The possible structures of the chemicals in principal peaks are shown. The ESI-MS cone voltage (170 V) was selected. Scanning was performed from *m*/*z* 100 to 1000 in 10 s, and several scans were summed to obtain the final spectrum, Figure S2: SDS-PAGE analysis of three purified and crude commercial metalloproteases. (A) Analysis of crude (Line 1) and purified (Line 2) MP; (B) Analysis of crude (Line 2) and purified (Line 1) DENIE-B LPS-P; (C) Analysis of crude (Line 2) and purified (Line 1) ViscozymeL.
