# Supplementary material for: Structure-Based Design and Synthesis of a New Phenylboronic-Modified Affinity Medium for Metalloprotease Purification
Source: Mar Drugs. 2016 Dec 27;15(1):5. doi: 10.3390/md15010005 (PMC5295225; doi:10.3390/md15010005)
Supplement: Supplementary file 1 [file marinedrugs-15-00005-s001.docx]

Supplymentary Materials: Structure-Based Design and Synthesis of a New Phenylboronic-Modified Affinity Medium for Metalloprotease Purification

Shangyong Li, Linna Wang, Ximing Xu, Shengxiang Lin, Yuejun Wang, Jianhua Hao and Mi Sun


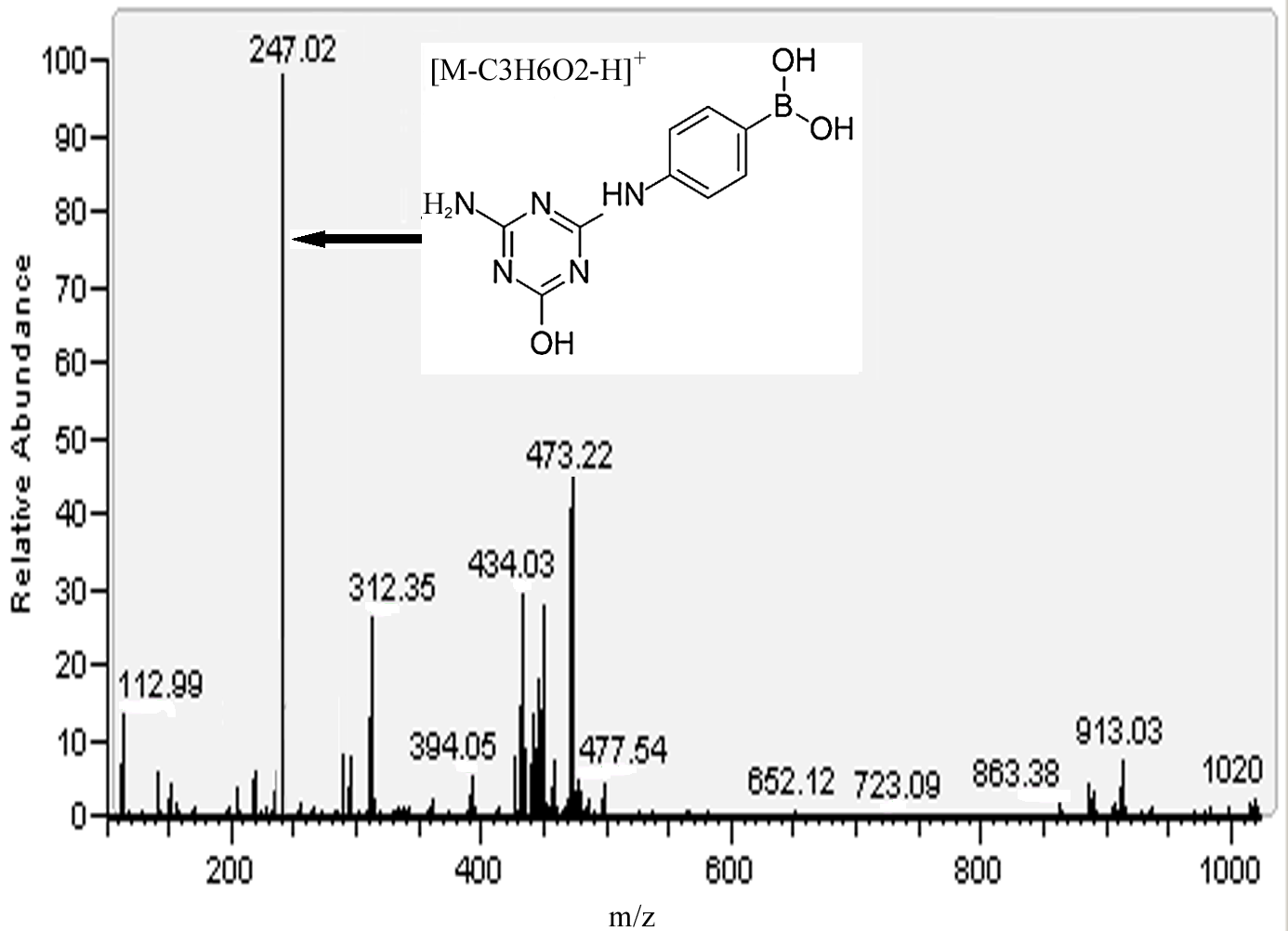


**Figure S1.** The ESI-MS analysis of the affinity ligand. The possible structures of the chemicals in principal peaks are shown. The ESI-MS cone voltage (170 V) was selected. Scanning was performed from *m*/*z* 100 to 1000 in 10 s, and several scans were summed to obtain the final spectrum.


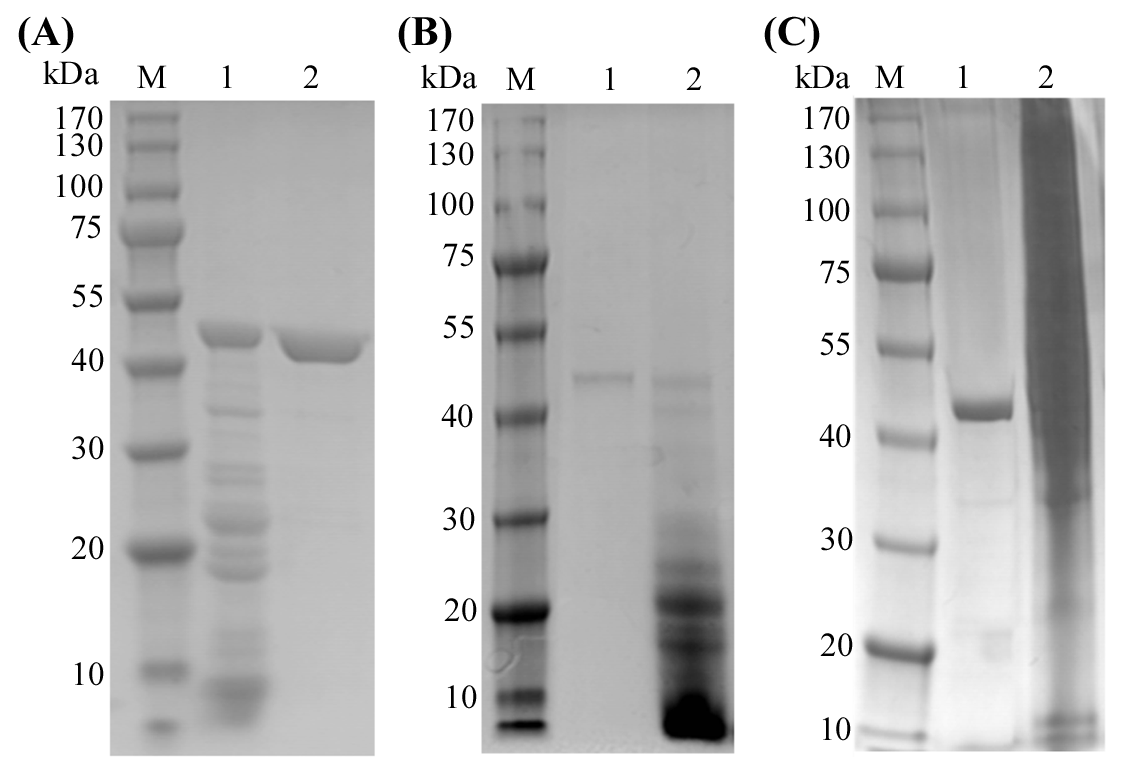


**Figure S2.** SDS-PAGE analysis of three purified and crude commercial metalloproteases. (**A**) Analysis of crude (*Line* 1) and purified (*Line* 2) MP; (**B**) Analysis of crude (*Line* 2) and purified (*Line* 1) DENIE-B LPS-P; (**C**) Analysis of crude (*Line* 2) and purified (*Line* 1) ViscozymeL.
